# A Novel Pot-Economy Approach to the Synthesis of Triantennary GalNAc-Oligonucleotide

**DOI:** 10.3390/molecules29245959

**Published:** 2024-12-17

**Authors:** Artem Evgenievich Gusev, Vladimir Nikolaevich Ivanov, Nikolai Andreevich Dmitriev, Aleksandr Viktorovich Kholstov, Vladislav Aleksandrovich Vasilichin, Ilya Andreevich Kofiadi, Musa Rakhimovich Khaitov

**Affiliations:** NRC Institute of Immunology FMBA of Russia, 115552 Moscow, Russia; gusev.artem.evgenievich@gmail.com (A.E.G.); nikolai.dmitriev@chemistry.msu.ru (N.A.D.);

**Keywords:** oligonucleotides, phosphoramidite, GalNAc, N-acetylgalactosamine, siRNA, L96, pot-economy, green chemistry approach

## Abstract

N-Acetylgalactosamine (GalNAc) is an efficient and multifunctional delivery tool in the development and synthesis of chemically modified oligonucleotide therapeutics (conjugates). Such therapeutics demonstrate improved potency in vivo due to the selective and efficient delivery to hepatocytes in the liver via receptor-mediated endocytosis, which is what drives the high interest in this molecule. The ways to synthesize such structures are relatively new and have not been optimized in terms of the yields and stages both in lab and large-scale synthesis. Another significant criterion, especially in large-scale synthesis, is to match ecological norms and perform the synthesis in accordance with the Green Chemistry approach, i.e., to control and minimize the amounts of reagents and resources consumed and the waste generated. Here, we provide a robust and resource effective pot-economy method for the synthesis of triantennary GalNAc and GalNAc phosphoramidite/CPG optimized for laboratory scales.

## 1. Introduction

Synthetic oligonucleotides are a rapidly growing class of therapeutics drugs. More than a dozen synthetic oligonucleotides-based drugs are already in use [1,2]. The remarkable application of RNA-based therapies has already been reported for metabolic and rare diseases [3,4]. It is related to the ability of RNA-based therapeutics to interact directly with complementary mRNA, without the need to identify the target protein structure. This fact gives a great advantage in the use of oligonucleotides in comparison with small molecules or antibodies and helps in treating «undruggable» diseases [5]. Branches of oligonucleotides therapeutics such as RNA-based, including small interfering RNA (siRNA), antisense oligonucleotides (ASOs), and other RNA molecules, could offer significant potential. It is expected that new types of oligonucleotides such as microRNA and non-coding RNA (lnc) will be applied to other related problems solutions soon [6,7].

However, the application of oligonucleotides has some limitations in use. All of them have multiple negative charges on molecules surfaces and large molecular weight resulting in low cell uptake and potential therapeutics efficiency [8]. That is why the development of delivery strategies has become one of key problems in RNA-based therapies [9]. One of the applied strategies is the conjugation of the delivery molecules (small molecules, antibodies, and other molecules) to the RNA molecule.

Delivery molecule families such as N-acetylgalactosamine (GalNAc) are widely used as a delivery strategy for many types of agents like small molecules [10], glycopeptides [11], ASOs [12], and siRNA [13]. Due to the ability to stably interact with asialoglycoprotein (ASGPR), their conjugates are successfully used in the delivery of liver-targeted siRNA [13,14]. By conjugating GalNAc to nucleic acid molecules, it can be efficiently delivered into hepatocytes and elicit the corresponding biological response. This represents a reliable delivery strategy for nucleic acid-based therapeutics. It was also discovered and reported that, among various versions of GalNAc ligands, the GalNAc arranged in the triantennary approach was found to be the most suitable chemically improved and metabolically stable candidate [15]. As a result, a number of RNA-based therapeutics in conjunction with different GalNAc ligands were approved by the U.S. Food and Drug Administration (FDA) or have shown progressive results in various stages of clinical investigations [16]. As an example, an siRNA conjugated to the GalNAc ligand L96, called Inclisiran [17,18], directly inhibiting the synthesis of proprotein convertase subtilisin/kexin type 9 (PCSK9) in hepatocytes, leads to reducing low density lipoprotein (LDL) cholesterol [19,20].

As described above, it is obvious that the GalNAc family of ligands is a promising subject of interest for future oligonucleotide-based drug development and manufacturing. It is expected that these ligands could be synthesized in small- and large-scale loads during target drugs synthesis. The different paths for GalNAc synthesis ligands have already been reported [21,22]; among them, the examples of triantennary GalNAc ligands synthesis have also been mentioned [23,24,25,26]. From the synthetical point of view, the protocols described above are robust and reliable and grant goods results, but they can be sustainably challenged and optimized.

As an example, the synthesis path described in the related patents [25,26], proposed for medium or large-scale synthesis, consumes significant amounts of solvents granting relatively low yields. Moreover, some steps in these methods use non-eco-friendly and toxic reagents and solvents (like DCM or Pd/C). In further publications [23], the idea of decreasing consumption and waste and increasing yields was successfully pursued, but some toxic reagents were still presented.

Looking at this issue from the Green Chemistry [27] and pot-economy points of view, it becomes clear that there is room for optimization. The Green Chemistry approach could be taken in at least 4 of 12 principles. These principles are «Prevention», «Atom Economy», «Less Hazardous Chemical Synthesis», and «Safer Solvents and Auxiliaries». All these principles are related to the safety and economic optimization of the synthesis scheme. To increase the synergetic effect of the Green Chemistry perspective, a pot-economy approach [28] also could be applied as an optimization method to decrease a number of actions (separation, reconstitution, control, etc.), resulting in time and resource efficiency. This approach is gained through consequently conducting one or more synthesis stages in a reaction system subject to the possibility of joint implementation.

Here, we report a robust pot-economy approach for the synthesis of triantennary GalNAc and its phosphoramidite that can be used in automated oligonucleotide synthesis to introduce multiple GalNAc residues either to the 5-end or 3-end of native or chemically modified DNA and RNA oligonucleotides.

## 2. Results and Discussion

### 2.1. Original Methods’ Issues

As mentioned earlier, the methods described in previous works [23,24,25,26], although classical, occupying their place of honor within the framework of organic synthesis, are not without drawbacks. The synthesis of GalNAc-L96 was presented as part of a standard approach to synthesis.

**Figure 1:**
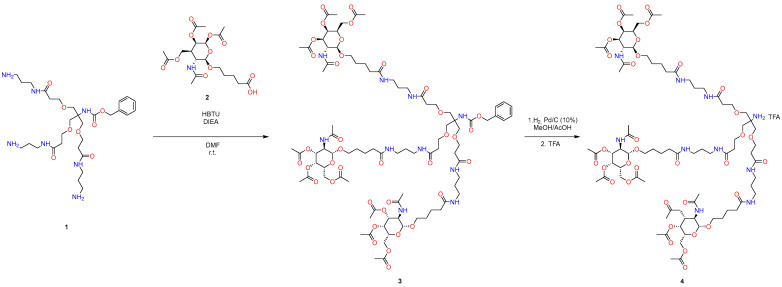
Standard approach to synthesize GalNAc-L96.

In the first stage, GalNAc-acid addition was realized using HBTU as a coupling reagent and using *Hűnig base*, and the desired product **3** was purified using column flash chromatography (Figure 1). As is known, this can be a challenge in the context of industrial production.

For the subsequent removal of the protective group from the amino group (Figure 1), standard hydrogenolysis conditions were used, which, although it is the most studied and widespread method, uses palladium in this process, which belongs to Class 1A [29], and therefore its widespread use in industry is rather frightening.

**Figure 2:**
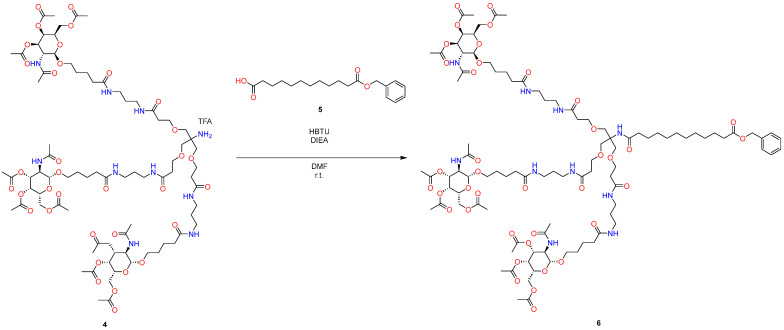
Standard approach to synthesize GalNAc-L96.

**Figure 3:**
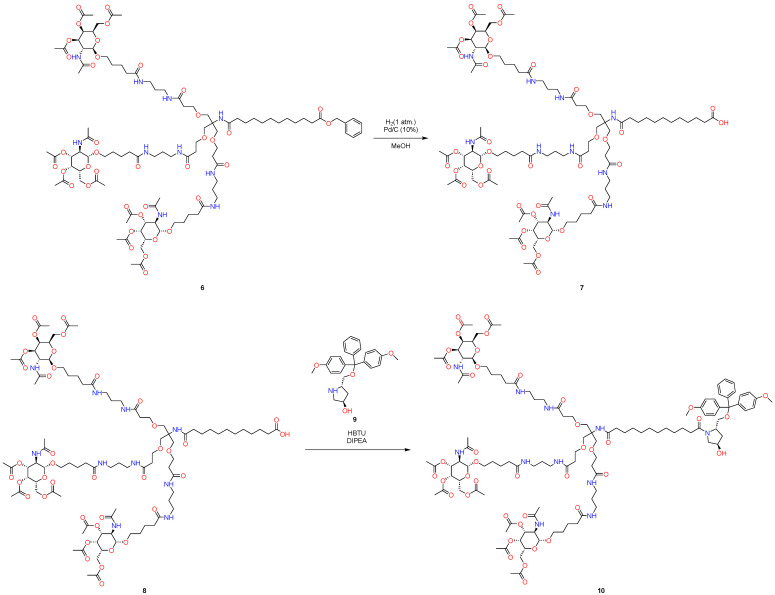
Standard approach to synthesize GalNAc-L96.

In this context, the issues raised earlier, such as the excessive reliance on solvents and the adherence to a standard procedure for the elimination of benzyl carbamate or benzyl protecting groups (Figure 2 and Figure 3), necessitate modifications and the exploration of innovative strategies that align with contemporary requirements.

At the last stage of GalNAc synthesis, nucleophilic addition was realized again by using HBTU as a coupling reagent with *Hűnig base*, and product **10** was again purified using column flash chromatography (Figure 3). As is known, this can be a challenge in the context of industrial production.

Thus, according to our opinion, there is room for improvement in the synthesis methods.

### 2.2. Alternative Synthesis Path

Therefore, within the framework of this work, a synthesis was proposed, which was designed based on the actual needs of the modern scientific community, since the significance of the target compound was described earlier, we must focus on the significance of the synthesis presented in this work. As part of the implementation of the synthesis scheme presented below, the main approaches that are necessary to assert that the synthesis meets the high standards of Green Chemistry have been implemented.

**Figure 4:**
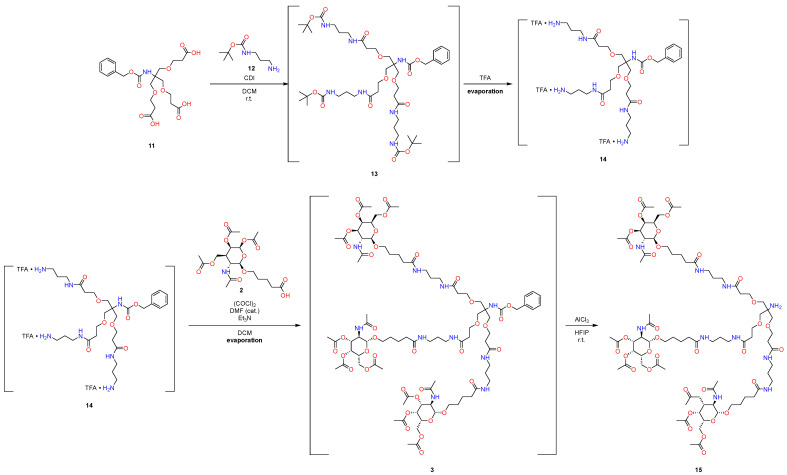
New approach to synthesize GalNAc-L96. First pot.

**Figure 5:**
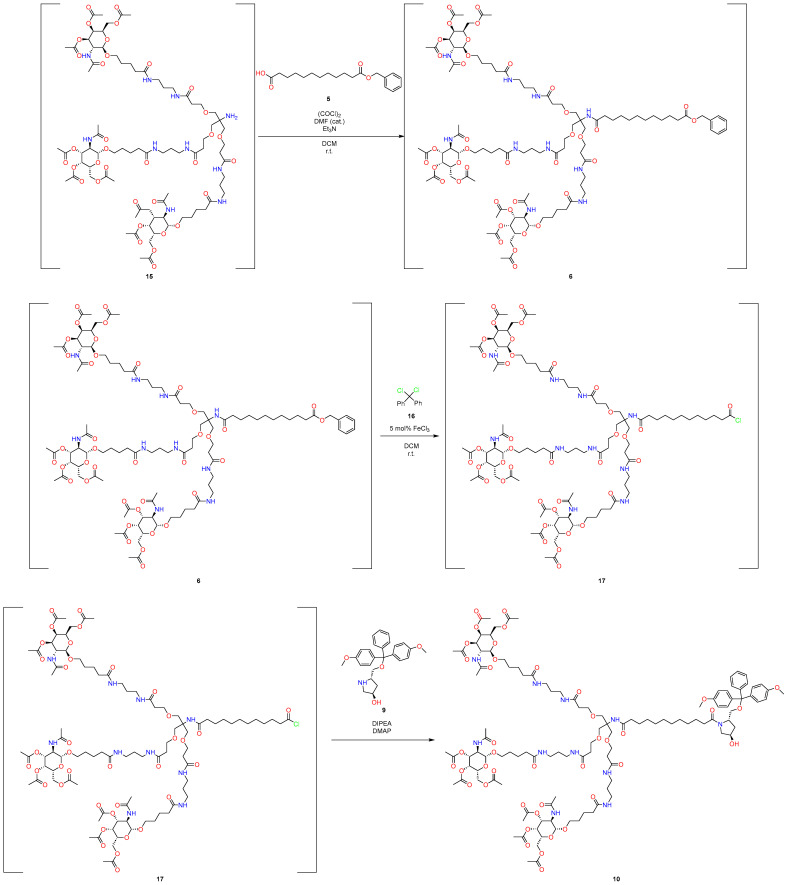
New approach to synthesize GalNAc-L96. Second pot.

First, a pot-economy synthesis was implemented as part of this work (Figure 4 and Figure 5). Namely, due to the need to use new conditions when removing protective groups, we had to divide the synthesis into two parts. This approach significantly reduces the number of solvents used and has all the advantages of the pot-economy approach.

Second, within the framework of the priority direction of the Green Chemistry approach, in particular when using the concept of pot economy, the proposed synthesis scheme was developed and successfully applied.

At the initial stages (Figure 4), product **13** was obtained from compounds **11** and **12** using CDI as a coupling reagent, with the subsequent removal of the protective group under standard conditions. To prepare the reaction mixture for the subsequent reactions, the excess TFA was removed by evaporation of the reaction mixture.

The subsequent connection was implemented within the framework of the standard protocol via acyl chloride. An alternative approach with sufficiently mild conditions was found for the removal of the benzyl carbamate protective group, which is described in [30], in which AlCl_3_ is used as LA, with the combined use of HFIP as a solvent; this approach allows selectively removing this protective group. This approach largely meets the standards of environmental friendliness of the materials used [31].

The next part of the synthesis (Figure 5), nucleophilic addition to acyl chloride was also realized; however, the cleavage of benzyl ether, as in the case of removal of the benzyl carbamate group, was carried out without the use of palladium and other standard conditions of hydrogenolysis. This approach using dichlorodiphenylmethane and catalytic amounts of iron (III) chloride was described in [32]; the key point in carrying out this reaction is that it allows cleaving benzyl ether and generating acyl chloride in situ under mild conditions, which, within the framework of this approach, reduces the synthesis by one stage, since it does not require generating acyl chloride separately for the subsequent nucleophilic addition of the amine. Flash chromatography was used to isolate the target product at the last stage.

The presented synthesis method allowed us to obtain the target product with a total yield of 61%. This reported result was the most successful L-96 synthesis result in a series of our experiments (small deviation with scales and reagents lots) with the average yield over 45%.

**Figure 6:**
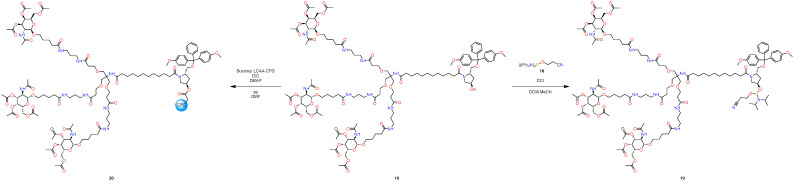
Synthesis of L-96 GalNAc phosphoramidite and L-96 GalNAc CPG.

The final part is a synthesis of L-96 GalNAc phosphoramidite and L-96 GalNAc CPG (Figure 6).

Third, as a symbiosis of pot-economy and Green Chemistry approaches we obtained a significant reduction in the total consumption of reagents and solvents and, consequently, a reduction in the possible wastes and an increase in the yields. We understand that such a direct comparison could be partially inappropriate due to differences in synthesis scales and purposes; so, we pay attention only to the rough numbers and trends.

We provide a very rough comparison (Table 1) related to the yields, use of rare metals, and the decrease in the consumption per synthesis of 1 g of compound **10** between publications [23,24,25,26]. We can see two opposite trends with an increase in the optimized synthesis yield with a decrease in the reagents used and the waste generated. In this comparison, the ratio between reagents used in the publications and the current methods is less than 10 times to 6 and 3 times. In addition, we can foresee the economy of reagents and solvents related to the purification and analytical steps due to the reduction in the number of stages via the pot-economy approach.

In future work, we would like to organize this method to scale up from 5 to 10 times with the same ratio of reagents and yields.

## 3. Materials and Methods

### 3.1. Materials

All reagents, unless otherwise stated, were obtained from either Sigma-Aldrich (Merck & Co., Inc., Rahway, NJ, USA) or ACROS (Acros Organics, Geel, Belgium) and were used as received. All solvents except for THF were used as received. THF was distilled over Na/benzophenone. Reactions were monitored by thin layer chromatography (TLC) carried out on Merck TLC (Merck & Co., Inc., Rahway, NJ, USA) silica gel plates (60 F254), using UV light for visualization and aqueous potassium permanganate or iodine fumes as developing agents. Flash column chromatography purifications were carried out using 60 silica gel (particle size 0.040–0.063 mm). All reactions were carried out in an air atmosphere unless otherwise stated.

All 1H and 13C NMR spectra were collected using a Bruker Avance 400 (Bruker Corporation, Billerica, MA, USA) instrument, with an operating frequency of 400 and 100 MHz, respectively, and calibrated using residual undeuterated chloroform (δH = 7.28 ppm) and CDCl3 (δC = 77.16 ppm) or undeuterated DMSO (δH = 2.50 ppm) and DMSO-d6 (δC = 39.51 ppm) as internal references. NMR data are presented as follows: chemical shift (δ ppm), multiplicity (s = singlet, d = doublet, t = triplet, q = quartet, m = multiplet, br. = broad), coupling constant in Hertz (Hz), integration.

IR spectra were recorded on a Thermo Nicolet IR–200 (Thermo Fisher Scientific, Waltham, MA, USA) in KBr or film. The mass spectra were recorded on an Agilent 6470 QQQ (Agilent Technologies, Santa Clara, CA, USA) mass spectrometer using JetStream electrospray ionization (ESI). High-resolution mass spectra (HRMS) were recorded on a Bruker maXis TOF (Bruker Corporation, Billerica, MA, USA) mass spectrometer using electrospray ionization (ESI) and a Bruker Microflex MALDI-TOF (Bruker Corporation, Billerica, MA, USA) mass spectrometer. The melting points were measured in open capillaries and are presented without correction. The known compound references with full analytical data are placed in the footnotes.

### 3.2. Methods

#### 3.2.1. 1st Pot

**Figure 7:**
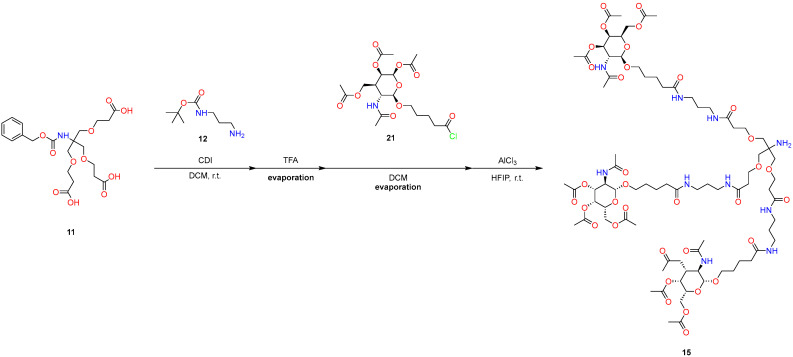
Part of synthesis in first pot.

1. The following operations are demonstrated in Figure 7.
2. To the solution of acid **11** (5.00 g, 10.61 mmol) in DCM (110 mL), CDI (1.72 g, 10.61 mmol, 1 eq.) was added by one portion, and the resulting mixture was stirred for 1 h.
3. Then, tert-butyl(3-aminopropyl)carbamate (**12**) (1.85 g, 10.61 mmol, 1 eq.) was added, and the resulting solution was stirred for 8 h.
4. After completion of the coupling reaction (TLC control), TFA (4.09 mL, 53.02 mmol, 5 eq.) was added, and the resulting solution was stirred for 24 h.
5. After completion of the cleavage of protecting groups, the reaction mixture was evaporated under reduced pressure to remove the excess TFA.
6. Then, the residue was dissolved in DCM (55 mL), and a solution of GalNAc acid chloride (**21**) in DCM (55 mL) (which was prepared from GalNAc acid (**2**) (14.23 g, 31.80 mmol, 3 eq.), oxalyl chloride (2.71 mL, 31.80 mmol, 3 eq.) and Et_3_N (4.43 mL, 31.78 mmol, 3 eq.) was added dropwise, by following the standard procedure using a catalytic amount of DMF). After stirring the reaction mixture for an additional 5 h, the resulting solution was concentrated in vacuo (55 °C, 1 h).
7. Then, to the solution of crude Cbz-amine (**3**) (20.43 g, 10.60 mmol) in HFIP (45 mL) was added AlCl_3_ (4.24 g, 31.79 mmol, 3 eq.) at room temperature, and the resulting suspension was stirred at the same temperature for 6 h. After the completion of the reaction, as observed by TLC and MALDI−TOF analysis, a partial amount of HFIP was recovered by using the downward distillation method.
8. The reaction mixture was diluted with DCM (100 mL). The reaction mixture was quenched with aqueous NaHCO_3_ (100 mL) and extracted with DCM (3 × 100 mL). The combined organic layers were washed with brine (100 mL), dried over anhydrous Na_2_SO_4_, filtered, and evaporated to give the 19.21 g crude desired product (**15**).

1H NMR (400 MHz, DMSO-d6); 1.33–1.54 (m, 14 H), 1.53–1.70 (m, 2 H), 1.70–1.83 (m, 9 H), 1.81–1.96 (m, 9 H), 1.93–2.06 (m, 12 H), 2.06–2.14 (m, 9 H), 2.19 (t, *J* = 7.06 Hz, 3 H), 2.27 (t, *J* = 5.32 Hz, 5 H), 2.99–3.10 (m, 9 H), 3.31–3.45 (m, 8 H), 3.47 (br. s., 1 H), 3.54 (t, *J* = 5.75 Hz, 7 H), 3.63–3.79 (m, 6 H), 3.78–3.96 (m, 5 H), 4.02 (s, 10 H), 4.49 (d, *J* = 8.50 Hz, 3 H), 4.88–5.07 (m, 5 H), 5.21 (d, *J* = 3.36 Hz, 3 H), 7.20–7.43 (m, 3 H), 7.72–8.09 (m, 5 H).

#### 3.2.2. 2nd Pot

**Figure 8:**
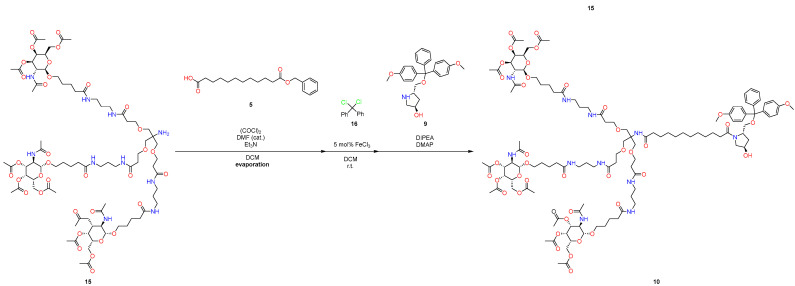
Part of synthesis in second pot.

1. The following operations are demonstrated in Figure 8.
2. The crude residue of compound **15** was dissolved in DCM (55 mL), and the resulting solution was added dropwise to the solution of 12-(benzyloxy)-12-oxododecanoic acid chloride in DCM (55 mL) (which was prepared from 12-(benzyloxy)-12-oxododecanoic acid (**5**) (3.40 g, 10.60 mmol, 1 eq.), oxalyl chloride (902.92 µL, 10.60 mmol, 1 eq.) and Et_3_N (1.48 mL, 10.60 mmol, 1 eq.), by following the standard procedure using a catalytic amount of DMF). After stirring the reaction mixture for an additional 5 h, the resulting solution was concentrated in vacuo (55 °C, 1 h).
3. Then, the residue was dissolved in DCM (100 mL), and to the solution of benzyl benzoate (6), dichlorodiphenylmethane (**16**) (3.02 g, 12.72 mmol, 1.2 eq.) was added, followed by FeCl_3_ (86.1 mg, 530 µmol, 5.0 mol%), and the resulting mixture was stirred at room temperature for 2 h.
4. Then, (3R,5S)-5-[[bis(4-methoxyphenyl)-phenylmethoxy]methyl]pyrrolidin-3-ol (**9**) (5.34 g, 12.72 mmol, 1.2 eq.), DIPEA (2.77 mL, 15.90 mmol, 1.5 eq.), and DMAP (259.0 mg, 2.12 mmol, 0.2 eq.) were added to the reaction mixture and stirred for 8 h at room temperature.
5. The mixture was poured into water (150 mL) and extracted with DCM (2 × 100 mL). The combined organic layers were washed with brine (200 mL), dried over anhydrous Na_2_SO_4_, filtered, and concentrated.
6. The resulting residue was purified by flash column chromatography on silica gel with DCM-EtOH-Et_3_N (3%) as an eluent from 20:1 to 5:1 (5:1 R_f_ = 0.6) to afford 15.59 g. of the desired product (**10**). Total yield: 61%.

1H NMR (400 MHz, DMSO-d6); 1.49 (dd, *J* = 14.18, 7.46 Hz, 19 H), 1.56–1.67 (m, 2 H), 1.72–1.82 (m, 9 H), 1.87–1.92 (m, 9 H), 1.95–2.07 (m, 14 H), 2.06–2.15 (m, 7 H), 2.15–2.23 (m, 2 H), 2.23–2.38 (m, 8 H), 3.00–3.10 (m, 10 H), 3.10–3.24 (m, 11 H), 3.47–3.63 (m, 26 H), 3.66–3.78 (m, 13 H), 3.81–3.93 (m, 3 H), 3.96–4.06 (m, 7 H), 4.14 (br. s., 2 H), 4.38 (br. s., 1 H), 4.49 (d, *J* = 8.50 Hz, 2 H), 4.96 (dd, *J* = 11.22, 3.33 Hz, 2 H), 5.21 (d, *J* = 3.30 Hz, 2 H), 6.80–6.93 (m, 5 H), 7.01 (br. s., 1 H), 7.13–7.25 (m, 6 H), 7.25–7.37 (m, 6 H), 7.75–8.00 (m, 8 H).

#### 3.2.3. Chemical Modification for Further Oligonucleotide Synthesis

**Figure 9:**
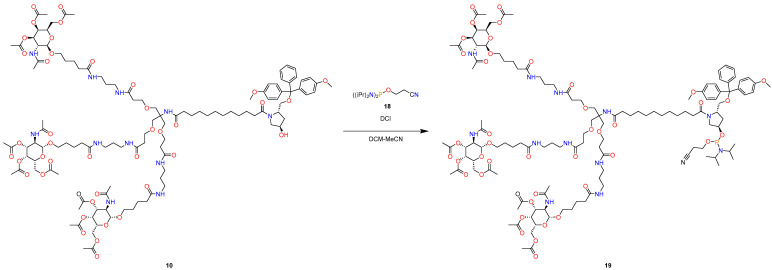
Synthesis of L-96 GalNAc phosphoramidite.

**Figure 10:**
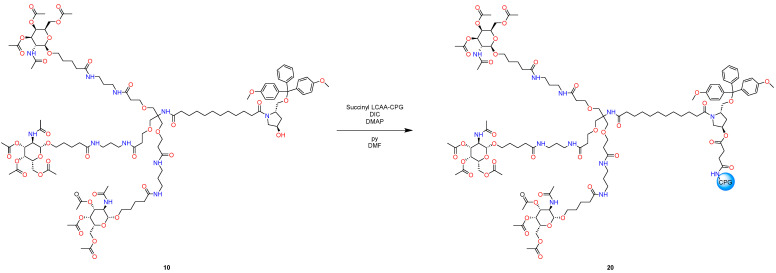
Synthesis of L-96 GalNAc CPG.

1. The following operations are demonstrated in Figure 9 and Figure 10.
2. Alcohol (**10**) (2.407 g, 1 mmol, 1 eq) was dissolved in DCM (20 mL), and to a clear solution in an argon atmosphere, 3-((bis(diisopropylamino)phosphaneyl)oxy)propanenitrile (**18**) (0.7525 g, 2.5 mmol, 2.5 eq) and DCI (0.118 g, 1 mmol, 1 eq) in 10 mL of MeCN were added. The reaction mixture was stirred in an argon atmosphere for 12 h. Then, the reaction mixture was poured into water, extracted with DCM, and dried over sodium sulfate. The organic phase was evaporated under reduced pressure. The crude material was precipitated with ethyl acetate and dried in vacuo to afford 2.1 g, a yield of 81% with 98.5% purity.

1H NMR (400 MHz, DMSO-d6); 1.04–1.16 (m, 10 H), 1.16–1.31 (m, 8 H), 1.35–1.57 (m, 15 H), 1.77 (s, 9 H), 1.85–1.92 (m, 9 H), 1.99 (s, 9 H), 2.01–2.05 (m, 2 H), 2.10 (s, 9 H), 2.23–2.33 (m, 6 H), 2.64–2.81 (m, 2 H), 3.04 (d, *J* = 5.38 Hz, 9 H), 3.38–3.43 (m, 3 H), 3.51–3.56 (m, 10 H), 3.87 (dt, *J* = 11.02, 8.92 Hz, 3 H), 3.96–4.08 (m, 8 H), 4.48 (d, *J* = 8.44 Hz, 2 H), 4.97 (dd, *J* = 11.22, 3.39 Hz, 2 H), 5.21 (d, *J* = 3.36 Hz, 2 H), 6.78–6.92 (m, 2 H), 7.13–7.24 (m, 3 H), 7.24–7.37 (m, 2 H), 7.74 (t, *J* = 5.53 Hz, 2 H), 7.83 (d, 3 H).

3. We charged (1.0 g, 0.4 mmol, 10 eq) compound **10** in a 25 mL round bottom flask and dissolved it in 8 mL of a 1:1 *v/v* pyridine-DMF mixture. Sequentially, we added (12 mg, 0.1 mmol, 2.5 eq) of DMAP, 1.0 g of succinylated LCAA-CPG 40 μmol/g, and diisopropylcarbodiimide (62 μL, 0.4 mmol, 10 eq), under stirring. We left the mixture at ambient temperature for 48 h and gently shook the mix daily. We gently shook the mix and immediately took a small portion, 150 μL, of the CPG suspension into a 1.5 mL tube and separated the CPG by decanting. We washed the CPG twice with 1 mL of DCM, twice with 2 mL of acetonitrile, and 5 times with 1 mL of diethyl ether. We dried the CPG in high vacuo for 1 h.
4. We accurately transferred ~15 mg of CPG **20** into a 25 mL flask and added 10 mL 5% TFA in DCM to it. After 15 min of exposure, we measured the absorbance of the mixture at 505 nm using a UV–Vis spectrophotometer, with DCM used as a blank, and we calculated the loading of GalNAc CPG using the following equation: L (μmol/g) = (V × 1000 × Abs)/78 × m, where m is the mass of **20** (in mg), Abs is the absorbance of the mixture at 505 nm, V is the total volume of the solution (mL), and 78 is the extinction coefficient of dimethoxytrityl cation (ml/μmol). The loading of the solid support **20** was calculated at ~32 μmol/g CPG. The yield of this step is over 80%. In addition, we set up the synthesis using a PolyGen oligo synthesizer, using 5 mg of the obtained CPG.

## 4. Conclusions

We have proposed an alternative approach to the synthesis of triantennary GalNAc, demonstrated on an L-96 ligand. The reagents were selected to meet the requirements of Green Chemistry, and the use of rare metals was avoided. The synthesis was designed from the pot-economy approach point of view to simplify it and reduce the consumption of reagents and materials, as well as the analytical and related procedures. The relative difference in total volumes or reagents and solvents used was from 10 to 3 times less in comparison with the previously described methods. In accordance with the presented data, we can draw the conclusion that the synthesis approach we proposed has a sufficient yield and can successfully compete with the previously described methods for the synthesis of similar compound groups. Due to its low impact on the environment and comparative simplicity, the proposed synthesis approach can considered for use on a future higher scale synthesis.

## Figures and Tables

**Table 1:** Comparative table of the current and reported methods.

Parameter	[24]	[26]		[25]		[23]		Current Approach
Yields, %	12	13		19		32		45 (best 61)
Rare metals used	Yes	Yes		Yes		Yes		No
		mL	Diff, times	mL	Diff, times	mL	Diff, times	mL
Total reagents used	NA	715	10	460	7	240	3	70
Solvents	NA	520	8	415	6	230	3	69
Basic reagents	NA	10	55	45	240	5	23	<1
Acidic reagents	NA	180	685	NA		1	6	<1

NA: Not applicable.

## Data Availability

The original contributions presented in this study are included in the article. Further inquiries can be directed to the corresponding author.
